# Protective Effects of Polysaccharides from *Sipunculus nudus* on Beagle Dogs Exposed to γ-Radiation

**DOI:** 10.1371/journal.pone.0104299

**Published:** 2014-08-05

**Authors:** Fengmei Cui, Ming Li, Yuejin Chen, Yuming Liu, Yin He, Dingwen Jiang, Jian Tong, Jianxiang Li, Xianrong Shen

**Affiliations:** 1 Department of Radiotoxicology, School of Radiation Medicine and Protection, Medical College of Soochow University, Suzhou, China; 2 Department of Health Toxicology, School of Public Health, Medical College of Soochow University, Suzhou, China; 3 Department of Protection Medicine, Naval Medical Research Institute, Shanghai, China; IIT Research Institute, United States of America

## Abstract

The aim of the study is to investigate the radioprotective effect of polysaccharide extract from *Sipunculus nudus* (SNP). Beagle dogs were randomly divided into the following six groups. Group-1: Un-treated and un-irradiated controls. Group-2: Exposed to a single acute dose of 2 Gy γ-radiation alone. Groups-3, 4 and 5: Oral administration of SNP at 50, 100 or 200 mg/kg body weight once a day for 7 days followed by a single acute whole body exposure to 2 Gy γ-radiation. The same doses of SNP were administered for further 27 days. Group-6: Positive controls treated with 1.6 mg/kg Nilestriol by gavage after radiation. Blood parameters including white/red cells and platelet counts, as well as hemoglobin level, were assessed every other day for 34 days (7 days before and 27 days of experiment). Serum separated from aliquots of the same blood sample was used to estimate enzyme activity of antioxidant superoxide-dismutase, and to determine levels of free radical, nitric oxide, hydroxyl and superoxide anion. At the end of the experiment, all dogs were euthanized to weigh the organs for organ co-efficient calculation. Pathological changes were assessed in the bone marrow. The results showed that the dogs exposed to γ-radiation alone exhibited a typical hematopoietic syndrome. In contrast, at the end of 27 days experiment, dogs received oral administration of SNP+γ-radiation showed: (i) a much improved blood picture as indicated by shorter duration of leucopenia, neutropenia, thrombocytopenia (platelet counts), as well as hemoglobin levels, (ii) significantly improved hematopoietic activity in the bone marrow, (iii) substantial decrease in nitric oxide levels, and notable increase in activity of antioxidant superoxide dismutase. The results suggested that oral administration of SNP in Beagle dogs was effective in facilitating the recovery of hematopoietic bone marrow damage induced by γ-radiation.

## Introduction

Damage to normal tissues is a consequence of both therapeutic and accidental exposures to ionizing radiation [1], [2]. Administration of medicine for radiation damage control is one of the most effective and direct approach to protect the injuries caused by ionizing radiation. Several compounds have been described to protect tissues from exposure to ionizing radiation [3], [4], but most of them have been shown to be highly toxic. As a result, there is an urgent need to develop compounds that may offer effective protection to radiation.

*Sipunculus nudus* belongs to the Sipuncula phylum and is widely available in sub-tidal zones and sea-beds of Beibu Gulf in China. The water extract from *Sipunculus nudus*, named as SNP, has long been used in traditional Chinese medicine to treat debilitating diseases caused by various pathogens, diabetes, tuberculosis, and carbuncles [5]. The main ingredient in *Sipunculus nudus* extract is polysaccharides [6]. The monosaccharides assembling the polysaccharides of SNP mainly comprise L-rhamnose, D-ribose, D-glucose, L-arabinose, D-mannose, D-galactose and D-glucose. The glycans from marine invertebrates are reported to contain polysaccharides which stimulate the immune function [7], [8], [9]. Several in vivo and in vitro studies suggested that polysaccharide intake could enhance immune functions [10], [11], [12], [13]. In many oriental countries, immunoceuticals containing polysaccharides such as lentinan, schizophyllan and krestin have been recommended to improve the general health of the population [14], [15]. The research have showed that SNP can stimulate the immunological function and have the effect of anti-fatigue in mice [16], [17]. Based on the biological effects of SNP that have been explored previously, the current study is aimed to assess its potential action in protection of Beagle dogs exposed to a moderate dose of γ(Gamma-ray)-radiation.

## Materials and Methods

### Ethic Statement

This study was carried out in strict accordance with the recommendations in the Guide for the Care and Use of Laboratory Animals of China. The animal study protocol was reviewed and approved by the Institutional Animal Care/User Ethical Committee of Soochow University (approval No. 20100909). All surgery was performed under sodium pentobarbital anesthesia, and all efforts were made to minimize suffering. The experimental procedures of all dogs were performed in accordance with the Regulations for the Administration of Affairs Concerning Experimental Animals approved by the State Council of People's Republic of China.

### SNP and Nilestriol

Water extract containing polysaccharides in *Sipunculus nudus* (SNP) (Lot No. 090328) was obtained from the Institute of Naval Medicine and stored in a dry place. The main ingredient in *Sipunculus nudus* extract is polysaccharides.

In this study, SNP screening was carried out according to the extraction process of polysaccharides [6]. Firstly, based on the examination of alkaline hydrolysis method, enzymatic method, alkaline hydrolysis combined with enzymatic method, protein removal method were studied and compared to obtain a better method. Secondly, the method was optimized by orthogonal design. The result of orthogonal design experiment showed that the order of the factors that affect the polysaccharide yield of the Sipunculus nudus is NaOH concentraion, raw material-liquid ratio, time, temperature. The optimized condition of extracting polysaccharides from the Sipunculus nudus is 1% NaOH, raw material-liquid ratio of 1∶4,60°C,4h.

The data from 3 pilot tests performed under the optimized condition showed that the method is stable. Specific procedure is as follows. Sipunculus nudus, weighed 45.5 kg, 43.0 kg and 41.8 kg respectively, were soaked overnight, washed, shredded by shears twice, and homogenized with 4 times the volume of water. 1% NaOH was added to extract for 4 hours in 60°C. The supernatant was collected after centrifuging at 9000 rpm for 30 min. The pH of supernatant was adjusted to 7.0, then to 4.0 by adding Trichloroacetic acid(TCA) with stirring. The supernatant was recollected after centrifuging at 9000 rpm for 30 min. 95% ethanol was added to the supernatant to the final concentration of 75%. The mixture was standing at 4°C for 12 h before 9000 rpm centrifugation for 30 min. The precipitation was collected, washed with 75% ethanol for 3 times, and dried. The content of SNP was measured and yield was calculated.

The content of protein was measured by Kjeldahl method using KDN-103F Kjeldahl apparatus(Shanghai Shine Jan Instruments Co. Ltd). The content of water was analyzed by drying method and the content of ash by ruling from appendix IX of ≪Chinese Pharmacopoeia≫ (published in 2005).

The components of polysaccharide were analyzed by gas chromatography (HP 6890 Plus, Hangzhou Kebo instrument Co. Ltd), HP-INNOWAX (30 m * 0.25 mm * 0.25 µm). 15mg of SNP was hydrolyzed with 10ml 3mol/L TFA at 120°C for 6h, concentrated and dried with methanol. The types of monosaccharide were determined by comparing the retention time(RT) of chromatographic peak with standard monosaccharide (Rhamnose, arabinose, xylose, mannose, glucose, galactose, inositol, fructose). Normalization method was used for quantitative calculation, shown as the percentage of the area of each chromatographic peak (Area%). The result suggested that the SNP was mainly composed of arabinose(7.91%)and glucose(79.32%). The rest 12% was shown as noise on the graph and can not be associated with the selected standards. They remain to be further studied.

The molecular mass of the polysaccharide was analyzed by SHIMADZU LC-20A RID-10A differential refractive index detector, with column phenomenexPolySep-GFC-P 4000 (300×7.8mm)mobile phase 0.71% Na_2_SO_4_. The RT of dextran standard samples with molecular mass of 2000000, 670000, 410000, 270000, 41100, 10000, 180 were determined as references. Under the same conditions, RT of SNP was also measured. The relative molecular weight of SNP was calculated as 385399.

As shown in the Table-1, the method applied was stable and repeatable. Both content and yield met the requirement.

**Table 1:** Results of extraction procedure and composition of extraction.

Batches	Weight of Raw Material(kg)	Yield(%)	Polysaccharides(%)	Protein(%)	Moisture(%)	Ash(%)
090328	45.5	3.5	86.8	2.3	2.5	0.5
090415	43.0	3.6	87.3	2.5	2.3	0.3
090425	41.8	3.3	85.4	2.4	1.6	0.5

Nilestriol (lot No. 20080601) was a present from Institute of Radiation and Irradiation Medicine, Academy of Military Medical Science and was used as a positive control drug.

### Experimental animals

Male Beagle Dogs weighing between 7 and 8 kg, aged between 7 and 8 months, were purchased from Fuzhou Zhenhe Technological Development Company, Ltd. which has the production license for experimental animals (SCXK (Min) 2007-0001). All dogs were housed in individual stainless steel cages in the animal facility of the Experimental Animal Center in Suzhou University which maintained 18–24°C temperature, 12-hour light/12-hour dark cycle. Each animal was fed with the standard dog food and provided tap water *ad libitum*. The cages were washed twice daily. All dogs were acclimatized to laboratory conditions for 7 days during which preventive measures were given as described below.

### Preventive measures

#### Infection prevention

Each dog was given intramuscular injection of 800,000 units of penicillin once a day when the basal peripheral white blood cell (WBC) count was less than 4x10^9^/L, and an additional intravenous injection of 1.0 g cefotaxime sodium once a day when the WBC count was less than 2x10^9^/L. After different treatments also (see below), penicillin was injected when the WBC count was less than 2×10^9^/L while no antibiotics were used when the WBC count was more than 4x10^9^/L.

#### Haemorrhage protection

Each dog received 1.0 g Yunnan Baiyao, which is a kind of hemostatic drug for treatment or prevention, oral administration, once a day when the basal peripheral platelet count was less than 1x10^11^/L. Each dog was also given 0.25 g vitamin C and 0.05 g aminomethylbenzoic acid dissolved in 20 ml physiological saline and an intravenous injection of 0.5 g etamsylate once a day.

#### Electrolyte supplement

During the experiment, dogs having severe vomiting or diarrhea were injected with sodium lactate ringer's injection and intravenous drip of glucose (50%) or oral rehydration salt II.

#### Intestinal problems

Each dog was injected with 160,000 units of gentamicin as well as oral administration of metronidazole of 0.4g once a day for 3 days before start of the experiment.

### Treatments

After 7 days acclimatization period, the dogs were randomly divided into six groups. Group-1: Un-treated and un-irradiated controls. Group-2: Exposed to a single acute dose of 2 Gy γ-radiation alone. Groups-3, 4 and 5: Oral administration of SNP at 50, 100 or 200 mg/kg body weight (low, medium and high doses, respectively) once a day for 7 days followed by a single acute whole body exposure to 2 Gy γ-radiation. The same doses of SNP were administered for further 27 days of recovery period. Group-6: Positive controls, treated with 1.6 mg/kg Nilestriol by gavage at day 1 before radiation and 5 h after radiation which is known to have a positive impact on hematopoietic system. Distilled water was used to obtain the required concentration of SNP. Each dog was also weighed every 3 days to adjust dose of SNP to be administered.

### Radiation exposure

The dogs in group 2–6 were anesthetized by intravenous injection of pentobarbital sodium (3%, 30 mg/kg), prostrated and whole body was exposed to γ-radiation by a single radiation with total dose 2.0 Gy at a dose rate 1.3 Gy/min using a ^6^°Co source located in the Medical Division of Suzhou University.

### Blood and Tissue collection

During the 34 days of the experiment (7 days before and 27 days of experiment), each dog was observed closely to record the appearance, activity, food intake, mucosal secretions in the mouth/penis, feces and body weights, and:

(1) From each dog, blood samples were collected on alternate days of the experiment (1, 3, 5, 7, 9, 11, 13, 15, 17, 19, 21, 23, 25 and 27 days) to obtain blood routine data, i.e., white and red cell counts, platelet counts and hemoglobin concentration. On days 3 and 13 after radiation, serum was separated from aliquots of blood samples to determine the levels of free radicals, viz., nitric oxide (NO), hydroxyl (OH) and superoxide anion (SA) as well as the activity of the antioxidant superoxide dismutase enzyme(SOD), using commercially available kits (Nanjing Jiancheng Bioengineering Institute).

(2) All dogs were sacrificed by femoral arteries bleeding and dissected on day 27 of the experiment after anesthetized. Each dog: (a) bone marrow smears were made on microscope slides to examine if there is any hyperplasia, (b) adrenal gland, epididymus, kidneys, liver, lung, spleen, thymus and testes were collected, their weights were recorded and organ coefficients were determined.

### Data analysis and assessment

All data were analyzed for significant differences, if any, between groups using the SPSS 17.0 statistical package. Repeated-measures data obtained from the peripheral blood were analyzed using ANOVA. Comparison among the groups was carried out by one-way ANOVA while *t*-test was used to compare with base values. Graphics were produced using Origin Pro 7.5 Software. The data in Tables 1–8 are mean ± standard deviation.

**Table 2:** White blood cells in Beagle dogs in different groups.

Days	Untreated	Model Con.	IR+SNP	IR+SNP	IR+SNP	Pos. Con.
	Con.	IR	Low	Moderate	High	IR+Nilestriol
		2 Gy	50 mg/kg	100 mg/kg	200 mg/kg	1.6 mg/kg
	(n = 4)	(n = 5)	(n = 5)	(n = 5)	(n = 5)	(n = 5)
Base	17.50±8.03	15.92±2.64	17.18±2.00	15.44±2.38	16.90±3.79	14.80±1.55
D1	13.93±1.93	9.10±1.79	11.04±1.74#	10.12±3.14#	10.53±3.16#	17.06±2.04*
D3	13.65±3.06	6.70±0.64	5.87±1.32	6.11±2.67	5.73±0.60	6.90±1.18
D5	13.90±2.39	5.80±1.26	5.90±0.97	6.09±1.96	5.43±0.87	5.98±1.25
D7	13.62±3.44	4.13±0.46	4.40±0.81	4.59±1.29	4.83±1.60	5.24±0.90
D9	14.68±4.09	3.14±0.63	3.68±1.01	4.19±1.47	3.43±1.17	4.80±1.25
D11	14.90±2.46	3.48±0.85	4.44±1.86	4.63±1.23	3.59±1.01	5.45±1.14*
D13	14.68±2.95	3.38±0.86	4.26±1.81	4.21±1.65	3.05±1.04#	5.50±1.28
D15	13.20±1.86	2.70±1.03	2.94±0.97#	3.16±0.96#	2.77±0.89#	4.89±1.67*
D17	14.63±1.53	2.56±0.87	3.04±0.63	3.02±0.55	3.02±0.95	4.02±0.83*
D19	15.33±0.90	2.38±0.97	3.03±0.68#	2.65±0.49#	3.05±0.98#	4.14±0.66*
D21	13.35±2.43	2.48±0.57	3.28±0.64#	3.47±0.24	4.49±1.66*	5.02±0.54*
D23	17.53±4.87	3.28±0.60	4.27±0.58	4.51±0.81	4.83±1.70	5.66±0.66
D25	14.45±1.84	3.96±1.68	5.20±1.01	6.32±0.84*	5.48±1.74	6.74±0.57*
D27	15.73±3.27	4.30±1.34	6.00±1.14	7.14±2.21*	6.61±2.68	8.35±1.47*
Nadir		2.21±0.76	2.61±0.84#	2.52±0.44#	2.44±0.74#	3.89±0.87*
Time to nadir (Days after radiation)		20.2±1.1	17.4±1.7	14.6±4.6*	16.6±3.3	17.8±1.8
Duration of leukopenia (Days)		16.0±6.5	12.4±5.2#	10.8±3.6#	12.0±6.3#	2.8±3.3*
Recovery level		27.1±7.3	35.4±8.0#	45.8±9.4*	40.8±21.3	57.0±11.7*

Note: Data are mean +/− standard deviation. The numbers are x10^9^/.

*: compared with model control, P<0.05;

# : compared with positive control, P<0.05. D1-D27 are the days of the experiment. After D3, WBC of all irradiated dogs were significantly lower than normal control, P<0.05. Nadir (x109/L) mean the lowest WBC. The recovery level (%) mean the ratio of the WBC on D27 to preradiation. Duration of leukopenia is defined as the days of white blood cell count (WBC) is lower than 4x10^9^/L.

**Table 3:** Neutrophils in Beagle dogs in different groups.

Days	Untreated	Model Con.	IR+SNP	IR+SNP	IR+SNP	Pos. Con.
	Con.	IR	Low	Moderate	High	IR+Nilestriol
		2 Gy	50 mg/kg	100 mg/kg	200 mg/kg	1.6 mg/kg
	(n = 4)	(n = 5)	(n = 5)	(n = 5)	(n = 5)	(n = 5)
Base	13.40±9.06	10.02±1.66	10.76±1.74	10.78±3.04	10.58±2.43	9.52±1.94
D1	10.05±2.91	7.38±1.88	9.48±1.92#	8.92±3.27#	9.32±3.31#	15.38±2.39*
D3	9.25±1.16	5.82±0.84	4.88±1.11	5.10±2.37	4.56±0.56	5.86±1.09
D5	10.53±3.33	4.76±0.96	4.86±0.86	5.20±1.72	4.38±0.66	4.52±0.72
D7	9.00±3.39	3.06±0.59	3.46±0.60	3.36±1.28	3.78±1.62	3.80±0.78
D9	9.58±2.99	2.20±0.70	2.64±0.80	3.12±1.61	2.18±0.81	3.54±1.09
D11	10.43±2.29	2.54±0.84	3.48±1.66	3.76±1.22	2.64±0.58#	4.40±0.87*
D13	9.23±2.98	2.52±0.80	3.30±1.54	3.38±1.73	2.26±0.86#	4.50±1.04
D15	8.83±2.02	1.74±0.98	2.02±0.65#	2.46±1.11	1.82±0.83#	3.82±1.48*
D17	9.48±1.71	1.30±0.66	1.74±0.39#	2.14±0.68	1.84±0.55	2.86±0.70*
D19	9.18±1.51	1.24±0.57	1.88±0.49#	1.90±0.42#	1.86±0.55#	2.96±0.64*
D21	9.28±1.58	1.50±0.56	2.12±0.42#	2.72±0.41*	2.80±1.03*	3.56±0.37*
D23	11.95±7.34	1.86±0.78	2.66±0.27	3.14±0.83	3.08±0.92	4.24±0.62
D25	9.63±1.20	2.50±1.05	3.72±0.68* #	4.90±0.83*	3.72±1.09* #	5.08±0.53*
D27	10.90±3.80	3.08±1.22	4.30±0.76	5.52±2.10	4.50±1.69	6.40±1.09*
Nadir		1.22±0.59	1.60±0.43#	1.66±0.32#	1.46±0.54#	2.68±0.61*
Time to nadir (Days after radiation)		17.8±1.8	17.8±2.3#	14.6±5.2	16.6±2.2#	11.0±4.7*
Duration of neutropenia (Days)		9.6±5.9	4.8±4.6	2.8±1.8*	5.6±5.4#	0.0±0.0*
Recovery level		30.6±9.5	40.9±9.9#	50.7±9.2*	42.8±16.1#	70.5±24.4*

Note: Data are mean +/− standard deviation. The numbers are ×10^9^/L.

*: compared with model control, P<0.05;

# : compared with positive control, P<0.05. D1-D27 are the days of the experiment. After D3, NEUT of all irradiated dogs were significantly lower than normal control, *P*<0.05. Duration of neutropenia is defined as the days of absolute neutrophil count (ANC) is lower than 2×10^9^/L. Nadir (x10^9^/L) mean the lowest Neutrophils. The recovery level (%) mean the ratio of the Neutrophils on D27 to preradiation.

**Table 4:** Thrombocytes in Beagle dogs in different groups.

Days	Untreated	Model Con.	IR+SNP	IR+SNP	IR+SNP	Pos. Con.
	Con.	IR	Low	Moderate	High	IR+Nilestriol
		2 Gy	50 mg/kg	100 mg/kg	200 mg/kg	1.6 mg/kg
	(n = 4)	(n = 5)	(n = 5)	(n = 5)	(n = 5)	(n = 5)
Base	357.0±99.5	377.0±78.5	373.8±36.7	342.8±99.1	302.8±70.2	322.8±40.4
D1	482.8±88.9	432.2±53.4	389.6±80.3	427.4±85.4	363.4±67.3	424.4±103.8
D3	406.8±134.7	396.0±62.4	342.2±74.2	359.6±125.3	336.6±89.7	384.6±140.7
D5	406.0±158.6	374.2±42.7	329.0±78.1	302.8±139.1	245.4±67.1	374.8±122.6
D7	389.3±107.2	237.6±43.4	215.0±86.2	213.3±88.8	127.3±56.4*	220.8±92.9
D9	421.3±81.8	109.3±29.1	93.1±42.9	111.5±61.5	68.8±26.6	87.4±50.6
D11	362.5±57.5	14.6±16.4	11.1±15.1	12.4±14.2	4.2±3.1	15.9±16.3
D13	329.0±40.1	4.3±1.9	5.1±1.4	7.6±5.0	5.2±1.5	13.3±15.9
D15	405.5±44.0	4.6±3.0	4.4±1.3	8.0±5.8	3.7±2.6	19.3±17.7
D17	406.8±85.0	7.7±1.6	8.6±2.5	23.8±17.0	5.4±4.9	31.3±19.7
D19	388.5±72.5	8.2±4.4	11.8±7.2	24.8±18.6	13.2±14.3	36.9±15.8
D21	374.0±72.0	17.1±16.6	20.9±23.9	26.0±19.0	13.7±14.9	44.4±22.0
D23	426.3±55.1	21.0±21.0	30.2±16.9#	39.2±22.9	24.4±21.0#	73.5±34.0*
D25	382.3±69.0	43.1±34.9	69.2±61.1	56.9±25.1	37.2±16.8	74.4±42.5
D27	413.3±157.4	68.7±52.6	104.0±79.7	73.0±36.7	55.6±22.6	94.0±58.7
Nadir		3.58±2.17	3.76±0.87	6.07±6.02	2.83±2.29	10.25±13.33
Time to nadir (Day after radiation)		12.6±1.7	12.6±1.7	12.6±1.7	14.2±2.3#	11.4±0.9
Duration of thrombocytopenia (Days)		10.8±3.6	8.0±2.8	6.8±5.2	11.6±3.3#	4.0±3.2*
Recovery level		20.4±18.4	27.1±19.0	22.0±11.1	18.4±5.2	30.0±18.7

Note: Data are mean +/− standard deviation. The numbers are ×10^9^/L.

*: compared with model control, P<0.05;

# : compared with positive control, P<0.05. D1-D27 are the days of the experiment. After D7, PLT of all irradiated dogs were significantly lower than normal control, *P*<0.05. Duration of thrombocytopenia is defined as the days of platelet count (PLT) is lower than 1×10^10^/L. Nadir (x10^9^/L) mean the lowest Thrombocytes. The recovery level (%) mean the ratio of the Thrombocytes on D27 to preradiation.

**Table 5:** Hemoglobin in Beagle dogs in different groups.

Days	Untreated	Model Con.	IR+SNP	IR+SNP	IR+SNP	Pos. Con.
	Con.	IR	Low	Moderate	High	IR+Nilestriol
		2 Gy	50 mg/kg	100 mg/kg	200 mg/kg	1.6 mg/kg
	(n = 4)	(n = 5)	(n = 5)	(n = 5)	(n = 5)	(n = 5)
Base	131.50±12.34	133.60±17.14	132.00±10.07	130.40±12.62	131.00±9.92	144.40±33.68
D1	134.75±14.64	135.80±11.56	138.40±3.05#	132.60±7.16	127.20±5.63	127.40±3.44
D3	144.25±8.96	133.40±12.05	138.60±11.67	127.20±9.52	123.20±10.52#	138.80±14.91
D5	138.50±15.97	131.00±9.95	136.60±8.85	135.00±8.80	124.60±12.32	132.80±6.61
D7	141.50±8.06	128.40±7.40	137.00±6.20	132.60±8.20	122.60±10.92	132.00±6.63
D9	137.25±16.17	120.00±6.16	128.20±3.35	128.80±11.48	116.78±14.83	125.20±6.80
D11	136.50±10.41	117.80±5.85	123.60±7.16	119.40±3.51	122.60±17.04	116.20±8.07
D13	133.25±5.12	115.40±5.98	117.40±6.77	120.00±6.44	116.54±12.93	121.40±6.77
D15	131.50±9.47	114.80±8.23	115.40±5.86	113.40±9.66	107.60±14.47#	122.00±6.78
D17	133.50±9.88	116.60±10.16	114.00±7.31	118.60±10.16	113.38±18.81	124.80±6.38
D19	141.75±8.42	113.20±9.01	114.80±4.38	115.20±9.42	112.56±18.13	120.40±7.09
D21	134.00±7.53	109.70±7.53	114.00±5.00	113.04±11.00	103.68±12.15#	123.60±7.23*
D23	147.00±9.56	114.40±11.80	117.00±6.04	115.00±11.20#	115.94±15.27#	130.40±7.64*
D25	138.25±11.09	113.20±12.01	116.40±7.64	117.40±10.57	113.28±14.80	125.60±7.77
D27	134.50±8.96	114.20±12.79	119.00±7.75	116.08±13.65	110.82±14.97#	127.40±7.83

Note: Data are mean +/− standard deviation. The numbers are g/L.

*: compared with model control, P<0.05;

# : compared with positive control, P<0.05. D1-D27 are the days of the experiment.

**Table 6:** Activity (%) in the bone marrow of Beagle dogs in different groups.

Groups	Promyelocytes	Neutrophils				Basophils	Polychromatics	Orthochromatics	Neutrophils∶Erythrocytes
		Myelocytes	Metamyelocytes	Band-form	Segmented-form				
Untreated Con. (n = 4)	1.63±1.31	3.50±1.00	8.63±3.33	15.38±3.25	19.13±5.19	3.50±0.00	14.38±3.88	19.50±5.07	1.46±0.52: 1
Model Con.: IR (n = 5)	1.75±0.50	2.50±0.58	6.25±4.52	8.25±2.72	11.75±5.89	2.63±0.75	19.00±6.96	36.75±11.62	0.59±0.17: 1
IR+SNP: 50mg/kg (n = 5)	1.25±0.65	2.10±0.96	4.20±2.97	11.90±2.82	11.10±7.85#	2.70±1.82	25.20±4.60* #	28.40±9.13	0.60±0.24: 1
IR+SNP: 100 mg/kg (n = 5)	1.90±0.96	3.00±0.94	4.90±2.10	12.40±3.03*	13.40±6.68	2.60±0.65	23.70±3.93	27.10±4.83	0.74±0.22: 1
IR+SNP: 200 mg/kg (n = 5)	2.50±0.87#	4.80±1.60* #	8.30±1.92	12.00±2.62	15.10±6.11	3.90±1.98#	20.20±1.82	27.20±9.07	0.76±0.43: 1
Pos. Con.: IR+ Nilestriol (n = 5)	1.10±0.65	1.70±0.57	6.50±1.46	12.00±2.03	20.90±3.32*	2.10±0.65	19.10±3.96	26.90±8.94	0.95±0.25: 1

*: compared with model control, *P*<0.05;

# : compared with positive control, *P*<0.05. Myelocytes∶Neutrophilic myelocytes; Metamyelocytes∶Neutrophilic Metamyelocytes; Band form: Band form neutrophils; Segmented form: Segmented form neutrophils. Basophilics∶Basophilic normoblast; Polychromatophilics∶Polychromatophilic Normoblasts; Orthochromatic∶Orthochromatic Normoblasts.

**Table 7:** Hyperplasia grades in the bone marrow in different groups of dogs.

Group	Active	App. Active	Inactive	Ext.Inactive	score	Hyperplasia Inhibition				Megakaryocyte decrease			
						+++	++	+	-	+++	++	+	-
Untreated Con. (n = 4)	1	3	-	-	3.8±0.5	-	-	-	4	-	-	-	4
Model Con.:IR (n = 5)	1	1	1	2	2.2±1.3	2	1	1	-	1	2	1	-
IR+SNP: 50mg/kg (n = 5)	1	3	1	-	3.4±0.9*	-	2	3	-	-	4	1	-
IR+SNP: 100 mg/kg (n = 5)	3	2			3.4±0.5*	-	1	3	1	-	3	1	1
IR+SNP: 200 mg/kg (n = 5)	-	5	-	-	4.0±0.0*	1	2	1	1	1	1	2	1
Pos. Con.: IR+Nilestriol (n = 5)	2	2	1	-	3.2±0.8	1	-	3	1	1	3	-	1

*: compared with γ-radiation alone, P<0.05. Standards and scores used to grade the activity in the bone marrow are as follows. Grades include five stages of extremely active, apparently active, active, inactive, and extremely inactive. Accordingly the ratio of mature erythrocytes to karyocytes is 1∶1, 10∶1, 20∶1, 50∶1, and 300∶1 and the score is 5, 4, 3, 2, and 1 respectively.

**Table 8:** Levels of free radicals and SOD in the serum in different groups of dogs.

Group	NO (µmol/L)		SOD (U)		OH-Scavenging(U/ml)		Superoxide-scavenging(U/L)	
	Day 3	Day 13	Day 3	Day 13	Day 3	Day 13	Day 3	Day 13
Untreated Con. (n = 4)	13.14±6.56	15.82±7.14	48.54±3.19	51.12±4.23	685.03±47.25	606.91±39.29	119.10±9.74	138.43±10.87
Molel Con.: IR (n = 5)	15.29±5.74	14.28±5.35	44.47±5.66	45.13±2.77	619.80±16.56	531.34±30.90	109.70±14.00	127.15±6.02
IR+SNP: 50mg/kg (n = 5)	13.53±3.88	5.31±2.83*	48.75±4.40	47.79±4.60	587.35±73.48	621.36±177.95	113.84±7.86	130.79±6.36
IR+SNP: 100 mg/kg (n = 5)	13.92±3.21	12.24±3.82	48.58±4.63	50.39±6.69	608.35±49.69	668.12±167.73	122.91±5.84*	135.42±20.73
IR+SNP: 200 mg/kg (n = 5)	12.16±7.61	6.94±2.54*	53.26±4.33*	55.84± 2.68*	622.98±62.32	555.66±40.35	127.72±10.98*	139.76±23.71
Pos. Con.: IR+Nilestriol (n = 5)	12.94±2.54	10.20±5.35	48.24±1.98	47.01±1.99	653.53±26.51	551.24±49.07	116.98±4.86	131.49±5.62

*: compared with γ-radiation alone, P<0.05.

## Results

### 1. Organ weights and organ co-efficients

There were no significant differences in the weights of adrenal gland, epididymus, kidneys, liver, lung, spleen, thymus and testes between and among the dogs in all 6 groups. Similar results were observed for organ-co-efficients (data not shown here).

### 2. White Blood Cells(WBC)

The data presented in Table-2 indicated that the dogs exposed to a moderate dose of 2.0 Gy γ-radiation showed a typical ‘time-related’ response. The WBC counts were significantly reduced by 57.1% of the base/original value on day 1 (p<0.05) with a further decrease to the minimum on day 19, and slowly recovered to 27.1% on day 27. The mean duration of leukopenia (WBC <4x10^9^/L) was (16.0±6.5)d, of which the longest is about 25 days.

Dogs which were administered 50, 100 and 200 mg/kg SNP showed a significant decrease in WBC on day 1 after the radiation, and went to minimum on days between 19 and 21 (2.65x10^9^/L and 3.28x10^9^/L). The mean duration of leukopenia was between 10 and 12 days, with the cell count reverted to 35.4%, 45.8% and 40.8% in 50, 100 and 200 mg/kg SNP dose groups at the end of the experiment, respectively. The recovery of the WBC count was faster in all SNP treated groups compared to that in γ-radiation alone group.

The WBC of the positive control dogs showed a transient and significant increase from the base value at day 1 (p<0.05) compared to the dogs in all other groups at the same time. The duration of leukopenia was significantly shorter, (2.8±3.3)d (p<0.05) and the WBC count reverted to 57% of the original value by the end of the experiment.

### 3. Neutrophils (Neut)

The data presented in Table-3 showed that the alterations in Neut in all groups of dogs were similar as those observed in WBCs.

The Neut count in dogs exposed to 2.0 Gy γ-radiation alone was decreased by 73.7% of the base value on day 1 and reached a minimum on day 19 (1.24×10^9^/L] followed by a slow recovery. The mean duration of neutropenia (<2x10^9^/L) was 23 days. The Neut counts reverted to only 30.6% of the original value at the end of the experiment.

Dogs which were administered 50, 100 and 200 mg/kg SNP had a significant decrease in Nuet (∼64%) on day 1 (p<0.05) and had minimum on days between 17 and 19 (1.74x10^9^/L and 1.90x10^9^/L). The mean duration of leukopenia was between 3 and 6 days. At the end of the experiment the Neut cell count reverted to 40.9%, 50.7% and 42.8% in 50, 100 and 200 mg/kg SNP groups, respectively. The recovery of the Neut count was faster in all SNP dose groups compared to those exposed to γ-radiation alone.

Positive control dogs treated with Nilestriol showed a transient increase to 161.6% of the original value on day 1 which was significantly higher than that in all other irradiated groups at the same time point (p<0.05) and then decreased slowly to a minimum (2.68x10^9^/L) on day 17. The Neut count reverted to 70.5% of the original value (significantly higher than that in all other irradiated dogs (p<0.05)) by the end of the experiment.

### 4. Platelets (PLT)

The data presented in Table-4 indicated no significant change in PLT count in dogs exposed to γ-radiation alone on day 1 while a progressive and significant decrease to a minimum (4.3x10^9^/L) was observed on day 13 (from the original value). The duration of thrombocytopenia (<9×10^10^/L) was 10 days. At the end of the experiment, the numbers reverted to only 20.4% of the original value.

The PLT counts in experimental dogs administered 50, 100 and 200 mg/kg SNP doses were not significantly changed on day 1 but there was a progressive and significant decrease between 13 and 15 days where the minimum counts reached between 3.76x10^9^/L and 7.6x10^9^/L. The duration of thrombocytopenia was 8, 7 and 12 days in dogs administered 50, 100 and 200 mg/kg SNP. At the end of the experiment, the PLT counts reverted to 27.1%, 22.0%, and 18.4% of their respective base value, respectively.

In dogs treated with Nilestriol, the PLT counts were not significantly altered until day 7 and then showed a decrease to a minimum 13.3 x10^9^/L on day 13 (p<0.05) followed by a progressive increase (94x10^9^/L) by the end of the experiment. The duration of thrombocytopenia was significantly shorter (4 days) (p<0.05) and the PLT count reverted to 30.0% of the original value by the end of the experiment which was significantly higher than that in all γ-irradiated dogs (p<0.05).

### 5. Hemoglobin (HGB)

The results were presented in Table-5. There was no significant change in HGB levels on day 1 in all groups of dogs. However, dogs exposed to γ-radiation alone showed a significant decrease (115g/L, p<0.05) in HGB level on day 15 with no further significant alteration by the end of the experiment.

The HGB level in dogs administered 50, 100 and 200 mg/kg SNP showed similar trend as in dogs which were exposed to γ-radiation alone: significant decrease between 13 and 17 days with no further significant alteration by the end of the experiment.

Positive control dogs treated with Nilestriol showed a small but non-significant decrease in HGB during the 27 days of experimentation.

### 6. Myelogram

#### 6.1. Hematopoietic activity in bone marrow

The observations on the activity of different cell types in the bone marrow in different groups of dogs were presented in Table-6.

Compared with un-treated/un-exposed animals, dogs exposed to 2 Gy γ-radiation alone showed a significant increase in poly- and ortho-chromatic erythrocytes while no significant changes were observed in basophils, neutrophil/erythrocyte ratio, pro-myelocytes and neutrophil cell series. However, dogs which were administered 50 mg/kg SNP+γ-radiation showed a significant increase in polychromatic erythrocytes (p>0.05) while the 100 SNP+γ-radiation showed an increase in proportion of band form neutrophil and 200 mg/kg SNP+γ-radiation showed an increase in neutrophils and myelocytes.

#### 6.2. Microscopic evaluation of hyperplasia

**Figure 1:**
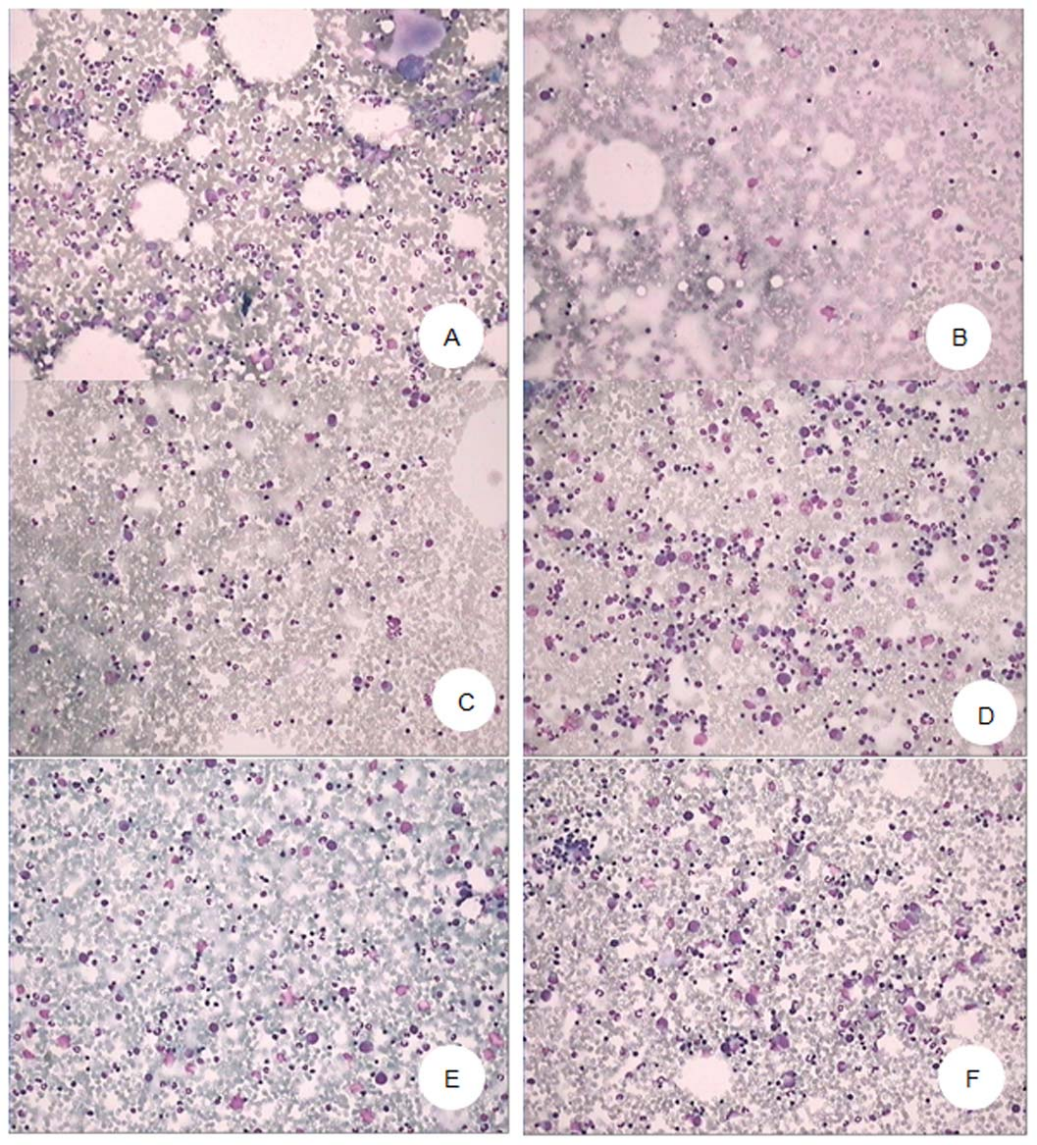
Bone marrow hyperplasia of Beagle dogs irradiated by 2.0^6^°Co γ ray, at Day 28 after radiation. A. Normal control (30^#^): Hyperplasia of karyocyte was apparently active. B. Model control (24^#^): Hyperplasia of karyocyte was inactive. C. Positive control (16^#^): Hyperplasia of karyocyte was active. D. Low dose of SNP (14^#^): Hyperplasia of karyocyte was apparently active. E. Moderate dose of SNP (8^#^): Hyperplasia of karyocyte was apparently active. F. High dose of SNP (1^#^): Hyperplasia of karyocyte was apparently active. (Giemsa ×200).

**Figure 2:**
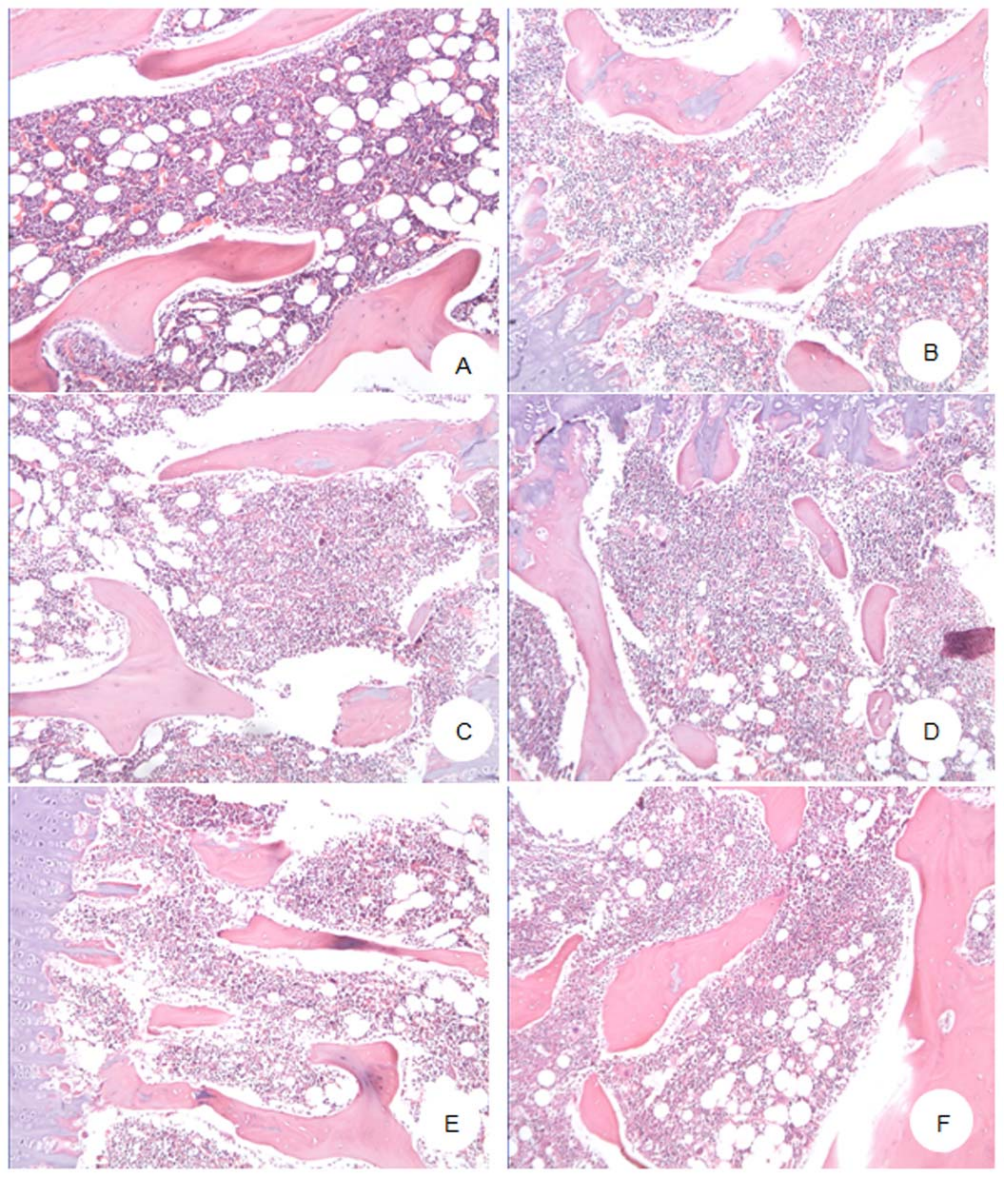
Pathology changes of Beagle dogs irradiated by 2.0^6^°Co γ ray, at Day 28 after radiation. A. Normal control (30^#^): Normal hemopoietic tissue hyperplasia in bone marrow and a large number of megakaryocyte. B. Model control (24^#^): Severe inhibition of hemopoietic tissue hyperplasia in bone marrow and rare megakaryocyte. C. Positive control (16^#^): Partial inhibition of hemopoietic tissue hyperplasia in bone marrow and a small number of megakaryocyte. D. Low dose of SNP (15^#^): Partial inhibition of hemopoietic tissue hyperplasia in bone marrow and a small number of megakaryocyte. E. Moderate dose of SNP (8^#^): Partial inhibition of hemopoietic tissue hyperplasia in bone marrow and a small number of megakaryocyte. F. High dose of SNP (2^#^): Partial inhibition of hemopoietic tissue hyperplasia in bone marrow and some megakaryocytes. (H.E×100).

The standard scores used to grade the hyperplasia as well as the results from microscopic evaluation of bone marrow smears were presented in Tables 7 (Figures 1 and 2). Four of the five dogs exposed to γ-radiation alone indicated scores of 2, 1, 1, 1 indicating extremely active, active hyperplasia, apparently active and inactive hyperactivity, respectively.

All 5 dogs which received 200 mg/kg SNP+γ-radiation had apparent hyperplasia. In 100 mg/kg SNP group, 3 and 2 dogs had active and apparently active hyperplasia, respectively. In 50 mg/kg SNP group, 1, 3 and 1 had active, apparently active and inactive hyperplasia, respectively.

Among the 5 positive control dogs treated with Nilestriol, 2, 2 and 1 had active, apparently active and inactive hyperplasia.

#### 6.3. Classification/grades of hyperplasia and megakaryocytes

The extent of hyperplasia was further classified into different grades (Table-7): severe inhibition (+++), moderate inhibition (++), slight inhibition (+) and normal (−). Megakaryocytes were graded as: severe decrease (+++), moderate decrease (++), slight decrease (+) and normal (−). The results presented in Table-6 indicated that the dogs exposed to γ-radiation alone showed inhibition of bone marrow hyperplasia and decrease in megakaryocytes while such changes were ameliorated in dogs which received SNP+γ-radiation and, the same was observed in positive control dogs injected with Nolestriol.

### 7. Changes in Free radicals and SOD level

The data were presented in Table-8. On day 3, there was a substantial increase in nitric oxide free radical level (15.3 mM/L) in dogs exposed to γ-radiation alone, as compared to that in untreated-un-exposed controls (13.1 mM/L). In dogs exposed to 50 and 200 mg/kg SNP+γ-radiation, the nitric oxide level was not affected until day 3 (∼13 mM/L) and then significantly reduced on day 13 (5-7 mM/L).

The SOD enzyme level was significantly higher in 200 mg/kg SNP+γ-irradiated dogs on days 3 and 13 (53 and 56 U) as compared with the dogs exposed to γ-radiation alone (p<0.05). Also, on day 3, the scavenging activity of superoxide anion radicals in dogs exposed to 100 and 200 mg/kg SNP+γ-radiation was significantly higher than that in dogs exposed to γ-radiation alone (p<0.05). The concentration of NO in animals treated with both low dose and high dose SNP groups were significantly lower than those in the model control at day 13 after radiation (p<0.05).

## Discussion

Exposure to ionizing radiation may result in tissue damage and has negative impact on health. The search for compounds that can reduce the deleterious effects of radiation are of interest in therapeutic radiation for cancers and in the setting of accidental or terrorism related exposures [18], [19]. Decades of preclinical and clinical research efforts have been spent with the aim of protecting normal tissue from radiation-induced damage, while there has only some limited success such as with amifostine [20]. The mainly reason is that many representative radiation protectors, radiation damage mitigators and radiation damage treatments also have significant toxicity. So other new compounds from natural plants were explored in recent years such as Curcumin, Orientin, Viciden, Ngella sativa and Podophyllum hovandrum [21], [22]. Currently many polysaccharides and polysaccharide–protein complexes have been isolated from mushrooms, fungi, yeasts, algae, lichens and plants have drawn the attention of researchers in biochemistry and medicine [23], [24]. In this endeavor, numerous polysaccharides from different biological origins have been investigated for immunomodulating activities and other functions [25], [26].

In our earlier investigations, we have used beagle dogs to investigate the toxic effects of SNP. By administration of 300 mg/kg SNP to the dogs, there were no notable adverse effects that could be observed (data not shown). In this study, we used doses of 50, 100 and 200 mg/kg body weight to observe the potential effects of SNP in protection of radiation.

One of the key deterministic consequences of radiation injury is the development of IR-induced clinical syndromes which depends highly on IR dose. It is widely known that relatively modest doses of IR exposures in humans elicit hematopoietic syndrome. In our research the results showed a much improved blood picture as indicated by a shorter duration of leucopenia, neutropenia, thrombocytopenia (platelet counts), as well as an increased hemoglobin levels. Human hematopoietic stem cells (hHSCs) were found to be exquisitely sensitive to radiation, responding with massive apoptosis to even modest doses of IR exposures [27]. Here the hematopoietic activity in the bone marrow was significantly improved. The damage to both the blood cells and the bone marrow induced by radiation were reverted and the recovery of erythroid and myeloid cells as well as hyperplasia in the bone marrow was promoted. These changes indicate that SNP have good effect of mitigating hematopoietic syndrome.

In addition to improvement of the blood picture, a substantial decrease in nitric oxide levels and a notable increase in activity of antioxidant superoxide dismutase were also observed, which indicated an increased potential to scavenge free radicals resulted from the radiation. Since oxidative stress via excessive generation of ROS appears to play a role in the development of radiation damage [28], it was speculated that superoxide dismutase (SOD) increased by SNP, may help mitigate effects of radiation. It's exciting that SOD mimetics have been proposed for prevention of radiation pneumonitis [29].

The greatest or most effective protection appears to be in the middle dose group of IR-dogs in this study. The most effective protection dose of SNP for IR-mice is 300mg/kg in our previous study. According to the rule of dose design of new drug's efficacy, the equal dose as the middle dose of IR-dogs at 100mg/kg was set from IR-mice studies at 300mg/kg (300mg/kg of effective IR-mice equaling to 100mg/kg of effective IR-dog according to the conversion of body surface area). It is possible that the greatest protection of some indicators appeared to be in the middle dose group. Also some results showed the protection in the high dose group.

All these changes contribute to the induction of moderate hematopoietic syndrome in Beagle dogs received 2 Gy γ-radiation [30]. The experimental results suggested that oral administration of SNP in Beagle dogs was effective in facilitating the recovery of hematopoietic bone marrow damage induced by γ-radiation.

Based on the data presented here, SNP compounds are of potential interest in the setting of radiation protection. The more important point is that SNP has no toxic effects on body. Mechanistic investigations are needed to explore the nature and extend to which SNP plays the role in radioprotection.
